# Myonuclear Domain Flexibility Challenges Rigid Assumptions on Satellite Cell Contribution to Skeletal Muscle Fiber Hypertrophy

**DOI:** 10.3389/fphys.2018.00635

**Published:** 2018-05-29

**Authors:** Kevin A. Murach, Davis A. Englund, Esther E. Dupont-Versteegden, John J. McCarthy, Charlotte A. Peterson

**Affiliations:** ^1^The Center for Muscle Biology, College of Health Sciences, University of Kentucky, Lexington, KY, United States; ^2^Department of Rehabilitation Sciences, College of Health Sciences, University of Kentucky, Lexington, KY, United States; ^3^Department of Physiology, College of Medicine, University of Kentucky, Lexington, KY, United States

**Keywords:** myonuclei, Type 2 fibers, muscle damage, muscle regeneration, Pax7-DTA

## Abstract

Satellite cell-mediated myonuclear accretion is thought to be required for skeletal muscle fiber hypertrophy, and even drive hypertrophy by preceding growth. Recent studies in humans and rodents provide evidence that challenge this axiom. Specifically, Type 2 muscle fibers reliably demonstrate a substantial capacity to hypertrophy in the absence of myonuclear accretion, challenging the notion of a tightly regulated myonuclear domain (i.e., area that each myonucleus transcriptionally governs). In fact, a “myonuclear domain ceiling”, or upper limit of transcriptional output per nucleus to support hypertrophy, has yet to be identified. Satellite cells respond to muscle damage, and also play an important role in extracellular matrix remodeling during loading-induced hypertrophy. We postulate that robust satellite cell activation and proliferation in response to mechanical loading is largely for these purposes. Future work will aim to elucidate the mechanisms by which Type 2 fibers can hypertrophy without additional myonuclei, the extent to which Type 1 fibers can grow without myonuclear accretion, and whether a true myonuclear domain ceiling exists.

## Introduction

Skeletal muscle is a unique tissue for many reasons, the most striking of which is that its cells are multi-nucleated (i.e., syncytial). Further, as skeletal muscle nuclei are post-mitotic, there is a reliance on the dedicated myogenic stem cell population, called satellite cells, to fuse into the syncytium for the purposes of myonuclear addition or possibly replacement. These properties make satellite cells essential for post-natal muscle growth and muscle fiber regeneration after injury (Seale et al., 2000; Lepper et al., 2011; McCarthy et al., 2011; Murphy et al., 2011; Sambasivan et al., 2011). Following the discovery of satellite cells (Robertson, 1960; Katz, 1961; Mauro, 1961), scientists postulated that a given myonucleus can only transcriptionally govern a fixed volume of cytoplasm, or “myonuclear domain” (Cheek et al., 1971; Hall and Ralston, 1989; Pavlath et al., 1989). This hypothesis was reinforced by early experiments in rodents that indicated load-induced hypertrophy was accompanied by myonuclear accretion (Schiaffino et al., 1976).

The myonuclear domain theory in skeletal muscle can be traced back to the work of Dr. Charles Epstein, who reported that cell size was directly proportional to gene dosage in polyploidy liver cells (Epstein, 1967). Incidentally, hepatocytes have a marked capacity for hypertrophy without DNA synthesis (Nagy et al., 2001; Miyaoka et al., 2012), and resident hepatocyte nuclei can transcriptionally support at least a doubling in cell size (Kim et al., 2000). The idea of a rigid nuclear domain is nevertheless ascribed to highly plastic, multi-nucleated skeletal muscle fibers and is pervasive and engrained within the context of skeletal muscle fiber hypertrophy. The controversy surrounding this idea is evidenced by a recent debate regarding the necessity of satellite cell-mediated myonuclear accretion for loading-induced hypertrophy (Egner et al., 2017; McCarthy et al., 2017), as well as recent studies supporting the existence of a rigid myonuclear domain during hypertrophy (Egner et al., 2016; Goh and Millay, 2017; Hindi et al., 2017; Moriya and Miyazaki, 2018; Randrianarison-Huetz et al., 2018). The purpose of this *Mini Review* is to provide perspective on myonuclear domain flexibility in skeletal muscle fibers during hypertrophy. We will discuss recent examples of myonuclear domain flexibility, specifically in fast-twitch Type 2 fibers of humans and rodents, and highlight the emerging role of satellite cells as key mediators of extracellular matrix remodeling during adult muscle fiber growth.

## Evidence for Myonuclear Domain Flexibility During Hypertrophy

**FIGURE 1:**
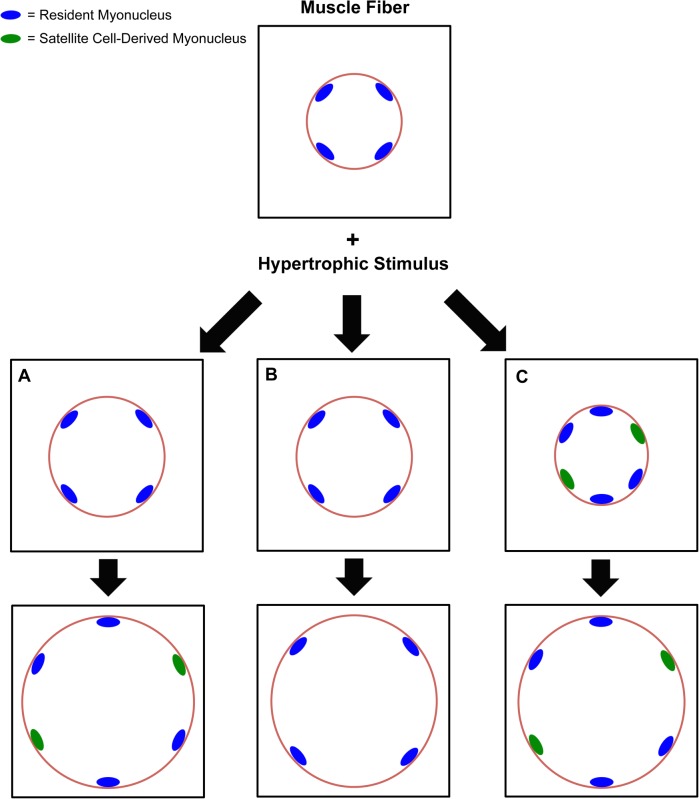
Flow chart describing the different perspectives on satellite cell-mediated myonuclear accretion and myonuclear domain expansion during Type 2 fiber hypertrophy across species. In scenario **(A)**, the myonuclear domain expands modestly until an upper limitation is reached, and then satellite cell-mediated myonuclear accretion ensues to support further hypertrophy. Evidence for this scenario is found in humans and rodents. In scenario **(B)**, satellite cell density increases in the absence of myonuclear accretion, and the myonuclear domain expands significantly or indefinitely as hypertrophy progresses. An upper limit in the myonuclear domain has not yet been identified. Evidence for this scenario is found primarily in rodent models, but significant hypertrophy without myonuclear accretion in humans has been reported. In scenario **(C)**, satellite cell-mediated myonuclear accretion precedes hypertrophy and is absolutely required for growth, implying that the myonuclear domain is tightly regulated. This scenario may apply to immature, growing skeletal muscle, but the evidence is limited in adult muscle.

Adult humans, mice, and rats have nearly identical myonuclear domain sizes (Liu et al., 2009). Numerous studies in humans and rodents report that satellite cell proliferation and myonuclear accretion occurs with skeletal muscle fiber hypertrophic growth (reviewed in Van der Meer et al., 2011a; Murach et al., 2018a). The often-observed incidence of large muscle fibers with a high proportion of myonuclei after training reinforces the notion that the myonuclear domain may expand modestly (Conceicao et al., 2018), but generally remains stable during hypertrophy (Scenario A in **Figure 1**). Muscle fiber hypertrophy and myonuclear accretion via testosterone supplementation further implies that myonuclear accretion directly contributes to adult muscle growth (Sinha-Hikim et al., 2003; Egner et al., 2013). Conversely, time-course studies in rats (van der Meer et al., 2011b) and humans (Kadi et al., 2004) show a substantial degree of loading-induced muscle fiber hypertrophy can occur prior to or in the absence of myonuclear accretion, resulting in significant expansion of the myonuclear domain.

Type 1 and 2 muscle fibers demonstrate differential hypertrophic plasticity under a wide variety conditions in humans (Luden et al., 2010; Murach et al., 2014, 2018b), and myonuclear domain flexibility during hypertrophy accordingly seems to differ by fiber type. Our laboratory (Fry et al., 2014b) and others (Herman-Montemayor et al., 2015; Damas et al., 2018) showed that the myonuclear domain of human Type 2 muscle fibers, which comprise ∼50% of muscle fibers in most muscles, is highly flexible. Herman-Montemayor et al. (2015) reported Type 2 fiber hypertrophy > 30% with non-statistically significant myonuclear accretion (9.5%, *P* < 0.10) and a 29% expansion of the myonuclear domain after resistance training in untrained women. These findings do not necessarily rule out the existence of a “myonuclear domain ceiling”, but do challenge the idea of a muscle fiber growth “threshold” beyond which myonuclear accretion is theoretically required to sustain hypertrophy (Kadi et al., 2004; Petrella et al., 2006; Conceicao et al., 2018). This threshold was derived most recently from correlations in human work and, while translational outcomes in humans are the ultimate goal, loss-of-function studies in mice are necessary to complement the indirect, correlative evidence gained in humans and to determine causality. It has also been proposed that differences between “low” and “high” hypertrophic responders to resistance training was explained by the extent of myonuclear accretion (Petrella et al., 2006, 2008), but this has recently been challenged in the literature by evidence showing myonuclear accretion is uncoupled from muscle fiber hypertrophy (Mobley et al., 2018). We acknowledge that baseline myonuclear density and/or fiber size could influence the requirement for myonuclear accretion during hypertrophy (Snijders et al., 2016), but it should be noted that identifying *bona fide* myonuclei on histological cross sections (the prevailing method) is somewhat subjective. The absolute number of myonuclei in a muscle fiber is dependent on the assessor’s interpretation of whether the central mass of a nucleus is inside or outside of a dystrophin border, which is highly contingent on the quality of staining and the experience of the technician. This ambiguity makes it difficult to compare absolute values across laboratories. The development of an antibody against the myonuclear marker PCM-1 may help resolve this issue (Winje et al., 2018).

Genetically modified mouse models provide overwhelming evidence for Type 2 fiber-specific hypertrophy in the absence of myonuclear accretion (Scenario B in **Figure 1**). Following conditional genetic deletion of satellite cells, our laboratory reports substantial hypertrophy in the plantaris muscle, primarily composed of Type 2 fibers, following synergist ablation surgery in adult mice (>4 months old) (McCarthy et al., 2011, 2017; Fry et al., 2014a, 2017; Kirby et al., 2016; Murach et al., 2017b, 2018a). Importantly, the lack of myonuclear accretion during hypertrophy does not negatively affect single muscle fiber contractile function (McCarthy et al., 2011; Fry et al., 2014a). Furthermore, resident myonuclei possess a significant transcriptional reserve that compensates for a lack of satellite cells during Type 2 fiber hypertrophy (Kirby et al., 2016). A maximal transcriptional rate for a given myonucleus that corresponds to a specific myonuclear domain size has yet to be identified. Manipulating signaling pathways that are central to muscle fiber size regulation (e.g., Myostatin, AKT, JunB), in the absence of muscle overload or damage, also induces significant hypertrophy without myonuclear accretion and provides further evidence for myonuclear domain flexibility in Type 2 muscle fibers (Welle et al., 2007; Amthor et al., 2009; Blaauw et al., 2009; Raffaello et al., 2010; Lee et al., 2012; Wang and McPherron, 2012; Omairi et al., 2016). Interestingly, when Type 2 fibers are genetically modified to be more oxidative, hypertrophy is associated with myonuclear accretion (Omairi et al., 2016); this dovetails with human data showing oxidative Type 1 fiber hypertrophy from exercise is accompanied by increased myonuclear density (Fry et al., 2014b). More work is needed to determine whether increased biosynthetic activity associated with the high metabolic demands of oxidative muscle fibers influences fiber type-specific requirements for myonuclear accretion during hypertrophy. Alternative non-surgical methods of inducing hypertrophy in muscles other than the plantaris in mice will likely be required to address this question.

## Limited Evidence for Prerequisite Myonuclear Accretion to Support Adult Muscle Fiber Hypertrophy

It is clear that the myonuclear domain is more flexible than previously appreciated, but some evidence suggests that myonuclear accretion is absolutely required for, and in fact precedes hypertrophy (Scenario C in **Figure 1**). Type 2 fibers in young mice (<4 months old) appear to have an absolute requirement for myonuclear accretion to mount a hypertrophic response (Guerci et al., 2012; Egner et al., 2016; Goh and Millay, 2017; Hindi et al., 2017; Randrianarison-Huetz et al., 2018) which, as noted above, is not the case in full-grown adult mice (McCarthy et al., 2011; Fry et al., 2014a, 2017; Kirby et al., 2016; Murach et al., 2017b). The evidence for myonuclear accretion and contraction of the myonuclear domain preceding hypertrophy is very limited (Bruusgaard et al., 2010). Additionally, significant myonuclear accretion ensues in response to non-hypertrophic stimuli such as heavy endurance training (Frese et al., 2015, 2016; McKenzie et al., 2016), which is antithetical to the position that myonuclear accretion drives hypertrophy. This is especially salient since myonuclear accretion is not necessary for oxidative adaptations to endurance training (Jackson et al., 2015), even in the highly-active diaphragm muscle (Murach et al., 2017a).

The effects of anabolic steroid usage provide circumstantial evidence that myonuclear accretion facilitates hypertrophy. Supraphysiological testosterone levels elicit hypertrophy in conjunction with myonuclear addition (Sinha-Hikim et al., 2003; Egner et al., 2013), which is compounded during heavy resistance training and pronounced hypertrophy (Kadi et al., 1999; Eriksson et al., 2005). However, androgen signaling can increase myogenic cell proliferation and differentiation *in vitro* and *in vivo* (Lee, 2002; Fu et al., 2012; Serra et al., 2012; Deane et al., 2013), making it difficult to tease out the contribution of testosterone-directed fusion versus inflammation/damage versus hypertrophy on myonuclear accretion. Recent evidence also suggests that an inflammatory environment alone causes myonuclear accretion in the absence of exercise training and altered muscle fiber size (Boutrup et al., 2018). We interpret these findings to mean that satellite cell fusion to muscle fibers *in vivo* can be mediated solely by the signaling milieu and uncoupled from muscle fiber size. During short-term muscle atrophy induced by hind limb suspension in mice, the myonuclear domain is dramatically reduced in size, further demonstrating the flexibility of the myonuclear domain (Bruusgaard and Gundersen, 2008; Bruusgaard et al., 2012; Jackson et al., 2012; Murach et al., 2018a). That is to say, if the myonuclear domain were truly rigid, one may expect myonuclear loss to consistently scale with atrophy in order to maintain the myonucleus-to-protein ratio, which would be consistent with the original “DNA unit” concept that is the foundation of the myonuclear domain theory (Cheek et al., 1971). Worth noting is that the duration of unloading, as well as the species and muscle under investigation, can produce differing results regarding myonuclear loss during unloading (reviewed in Murach et al., 2018a). Since atrophy and hypertrophy are distinct processes, more work is needed to determine whether prerequisite myonuclear accretion is required for regrowth after unloading in instances where myonuclei are lost during atrophy (Ohira et al., 1999; Siu et al., 2005; Dupont-Versteegden et al., 2006).

## Perspectives on the Early Satellite Cell Response to Resistance Exercise

Following severe muscle fiber injury, satellite cells mediate skeletal muscle regeneration via myogenesis and interactions with fibroblasts to coordinate proper extracellular matrix remodeling (Murphy et al., 2011). Resistance exercise in humans is not typically associated with muscle degeneration, but muscle injury, inflammation, and sarcolemmal damage may ensue (Damas et al., 2016). One hypothesis is that the magnitude of early satellite cell proliferation after unaccustomed resistance exercise reflects myonuclear accretion potential and predicts hypertrophic adaptation; however, the relationship to muscle fiber growth is unclear (Bellamy et al., 2014). Once muscle damage subsides, satellite cell proliferation after resistance exercise is attenuated (Murach et al., 2016; Damas et al., 2018), and is not predictive of the ∼16% Type 2 muscle fiber growth reported by Damas et al. (2018). Consistent with initial conjectures on early satellite cell proliferation with overload (Snow, 1990), it seems that muscle fiber damage and satellite cell niche disruption primarily dictates satellite cell responses to resistance exercise, and not hypertrophy *per se*. The relationship between exercise-mediated muscle fiber damage and satellite cell proliferation is underscored by greater satellite cell density after highly- versus minimally-damaging contractions in exercise-naïve men (Crameri et al., 2007; Hyldahl et al., 2014).

Under certain conditions, the propensity to fuse is an inherent property of activated satellite cells (Moss and Leblond, 1971). As such, it is conceivable that satellite cell-mediated myonuclear accretion observed with resistance training is simply a response to muscle fiber damage and the accompanying milieu, and not a requirement to maintain myonuclear domain size. In support of this hypothesis, Type 2 fiber myonuclear accretion occurs during 12 weeks of eccentric (i.e., damaging) but not concentric (i.e., minimally damaging) resistance training, despite similarly modest hypertrophic responses to both modes (Farup et al., 2014). The most strenuous exercise may induce the most damage, and subsequently the most myonuclear accretion. Worth mentioning is that the synergist ablation model of skeletal muscle overload used to induce hypertrophy in rodents can be rather severe and damage-inducing depending on how the surgery is conducted (Snow, 1990), which is an obvious drawback of the model that may affect translatability to humans. Nevertheless, muscle fibers can hypertrophy successfully without satellite cells using this extreme model, and in the absence of degeneration–regeneration (McCarthy et al., 2011). Interestingly, our laboratory recently showed that the early satellite cell proliferative response to mechanical loading is crucial for proper extracellular matrix remodeling during hypertrophy (Fry et al., 2017). Instead of myonuclear addition to muscle fibers, we suggest that the critical role for activated satellite cells in response to a hypertrophic stimulus is to participate in extracellular matrix remodeling which ulitmately facilitates growth, at least in Type 2 fibers (Fry et al., 2014a, 2017; Murach et al., 2018a).

## Conclusion

The role of satellite cells during hypertrophy extends beyond myonuclear accretion. Significant myonuclear domain flexibility is apparent in human and rodent muscle fibers during growth, which is most evident in Type 2 fibers. Satellite cells play an important role in the hypertrophic process, and we cannot rule out that myonuclear accretion may be necessary for hypertrophy under some conditions. However, we contend that satellite cell proliferation with loading, particularly in early phases but potentially throughout training, is likely for the purposes of supporting muscle repair and extracellular matrix remodeling, and not necessarily a precursor to fusion for augmenting transcriptional capacity during adult muscle fiber hypertrophy. The critical function of satellite cells in regulating the extracellular environment, but not necessarily driving growth via myonuclear accretion, can be leveraged to help guide therapeutics aimed at preserving or enhancing muscle mass and function. More work is needed to determine the upper limit of myonuclear domain flexibility and mechanisms regulating transcriptional reserve capacity, as well as the extent to which Type 1 fibers can grow without myonuclear accretion.

## Author Contributions

The manuscript was written by KM and DE, and the figure was created by KM and CP. All authors contributed intellectually and participated in the revising of the manuscript.

## Conflict of Interest Statement

The authors declare that the research was conducted in the absence of any commercial or financial relationships that could be construed as a potential conflict of interest.
